# Crystallization Selectivity of Ribavirin Solution and Amorphous Phase

**DOI:** 10.3390/molecules28176320

**Published:** 2023-08-29

**Authors:** Fuying Li, Shiying Chen, Haoxin Hu, Chengfeng Liang, Shiyu Sun, Can Jin, Fenghua Chen

**Affiliations:** 1 Fujian Provincial Key Laboratory of Resources and Environment Monitoring & Sustainable Management and Utilization, Sanming University, Sanming 365004, China; afu198207@163.com (F.L.);; 2 Department of Engineering Technology Management, International College, Krirk University, Bangkok 10220, Thailand; 3 College of Chemistry and Materials Science, Fujian Normal University, Fuzhou 350007, China; 4 Torch High Technology Industry Development Center, Ministry of Science and Technology, Beijing 100045, China

**Keywords:** ribavirin, crystallization selectivity, solution, amorphous phase, solvate, mid-frequency Raman difference spectra

## Abstract

Crystallization selectivity is an important principle in polymorph control. Ribavirin Form I, Form II, DMSO solvate, and amorphous ribavirin are prepared, and the short-range order similarities between these solid forms and ribavirin aqueous solution and DMSO solution are compared via mid-frequency Raman difference spectra (MFRDS). The crystallization process from amorphous ribavirin to Form I and from solution to amorphous phase is explained. Reasons for the difficulty in preparing the DMSO solvate are proposed. The rationale provided for the crystallization selectivity provides a foundation for the synthesis of metastable phases with a robust and convenient method.

## 1. Introduction

A broad definition of drug polymorphism includes molecular polymorphs, amorphous phases, hydrates and solvates, salts, and cocrystals, while a strict definition only includes crystal structures composed of the same molecules, atoms, or ions [1]. Polymorphism is a fundamental consideration in drug crystallization, and polymorph control is an important factor in drug safety and efficacy. Different crystal structures can confer different physical chemical properties, such as solubility, dissolution rates, and stability, and lead to different biological utilization [2]. For Biopharmaceutics Classification System (BCS) II and IV drugs, a low effective blood drug concentration is often caused by the low solubility of a drug polymorph, and it is necessary to find polymorphs with better solubility. A metastable drug polymorph can have serious drawbacks due to high solubility and phase transition during storage into a more stable, ineffective form, which may not be clinically suitable. Many technologies have been developed to distinguish drug polymorphs, including powder X-ray diffraction (PXRD), differential scanning calorimetry (DSC), Fourier transform infrared spectroscopy (FT-IR), Raman spectroscopy (Raman), solid–state nuclear magnetic resonance (ss-NMR), ultraviolet–visible spectroscopy (UV–Vis), hot-stage microscopy, small- angle X-ray scattering (SAXS), neutron scattering, and flow-driven spectral chaos (FSC) [3,4,5,6].

The control of molecular polymorphisms has been studied thoroughly in the laboratory and in industrial production, and selective crystallization processes are observed in many different systems [7,8], such as solution crystallization, melt crystallization, amorphous phase recrystallization, solvate desolvation, and evaporative crystallization [9,10]. For example, it is easy to obtain the metastable inosine α form from aqueous solution or the stable inosine β form from dimethylsulfoxide (DMSO) solution [11]. Amorphous guanine has a tendency to recrystallize into the metastable anhydrous guanine β form in biominerals and in the laboratory [12,13], and amorphous calcium carbonate can have different short-range orders, such as calcite or vaterite [14]. The product of guanine monohydrate dehydration is a framework of guanine monohydrate [15], and the dehydration product of inosine dihydrate is the metastable inosine α form [16]. However, theoretical explanations of selective crystallization processes are still somewhat inadequate.

The first step in crystallization is nucleation. In classical nucleation theory (CNT), monomers such as molecules, atoms, or ions in a supersaturated condition coalesce into a nucleation cluster, and then additional monomers attach one by one to form a crystalline phase [17]. The molecular arrangement in the nucleation cluster determines the polymorph. CNT nucleation is suitable to describe a process via monomer-by-monomer addition.

However, the crystallization process can also apply to the association of multi-ion complexes, oligomeric clusters, crystalline or amorphous nanoparticles, and monomer rich liquid droplets [18], for example. Non-classical nucleation theory (NCNT) was proposed to describe crystallization processes with these non-monomer additions. In the field of molecular crystals, the hypothesis that a certain molecular assembly in solution induces a polymorph of similar assembly structure was proposed to explain solution crystallization selectivity [3,4]. However, this theory cannot explain all crystallization processes, and the correlation between the solution associate and crystal synthon sometimes cannot be established [3,4]. We believe that the molecular assembly theory can explain the process from the non-monomer intermediate phase (assemblies) to crystals, but that it fails to explain the relationships between the monomer and crystal or monomer and non-monomer intermediate phase.

Standard analysis techniques that relate to monomer assembly and crystals include FTIR, Raman, NMR, and neutron scattering. However, they are inadequate in correlating all the related phases at the same time and establishing similarities among these phases. Our group has proposed mid-frequency Raman difference spectroscopy (MFRDS) to solve this problem and has established phase relationships to explain the selective crystallization behaviors of inosine and simvastatin [11,19]. It is still necessary, however, to test the power of MFRDS and identify new selective crystallization processes for well-researched molecules based on MFRDS results.

Ribavirin (C_8_H_12_N_4_O_5_, 1-β-D-ribofuranosyl-lH-1, 2, 4-triazole-3-carboxamide, CAS: 36791-04-5, Figure 1) is a broad-spectrum nucleoside antiviral drug and was recommended in the Interim Guidance for Diagnosis and Treatment (Seventh Edition) of COVID-19 [20]. The molecular structure of ribavirin contains flexible ribofuranosyl and rigid triazole structures. The flexible part endows ribavirin with rich conformational potential and its many hydrogen-bonding units induce complex intermolecular interactions. These factors favor the formation of polymorphs and amorphous phases, as is the case for guanosine and inosine [11].

**Figure 2:**
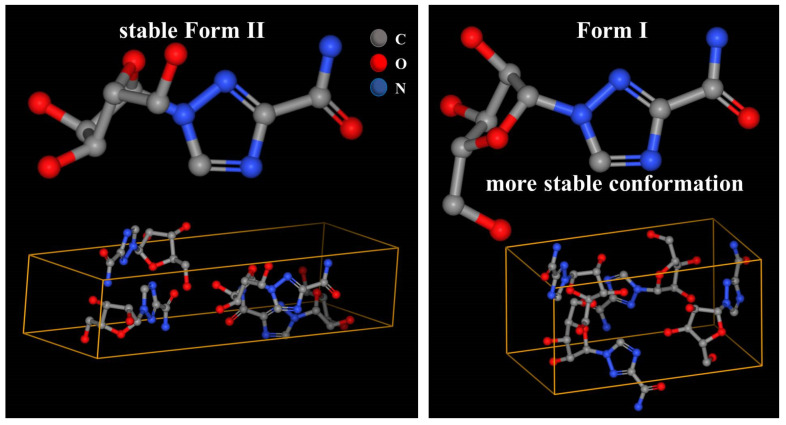
Molecular crystal structures of ribavirin Form II and Form I (public data from the Cambridge Crystallographic Data Centre, CCDC, www.ccdc.cam.ac.uk, accessed on 25 April 2023).

Anhydrous ribavirin has two polymorphs (stable Form II and metastable Form I, Table 1) and one amorphous phase. The conformations are quite different in Form II and Form I (Figure 2), and the conformational energy of Form I is significantly lower [21,22]. The single crystal of ribavirin DMSO solvate has been reported (Table 1) [23] but has not been published in the Cambridge Crystallographic Data Centre (CCDC). While the main synthesis methods of ribavirin solid forms are listed (Table 2), the selective crystallization of ribavirin solution, the recrystallization of amorphous ribavirin, and the desolvation of ribavirin DMSO solvate have not been explained.

In this work, the crystallization selectivity of ribavirin solution and the amorphous phase are studied by MFRDS. Selective crystallizations from amorphous ribavirin to Form I and from aqueous solution to amorphous ribavirin are effected and explained in depth. Furthermore, reasons for the poor reproducibility of ribavirin DMSO solvate are proposed. In general, MFRDS is further demonstrated as a theoretical foundation for the preparation of amorphous and metastable phases of organic molecules.

## 2. Results

### 2.1. Preparation of Ribavirin Polymorphs

**Figure 3:**
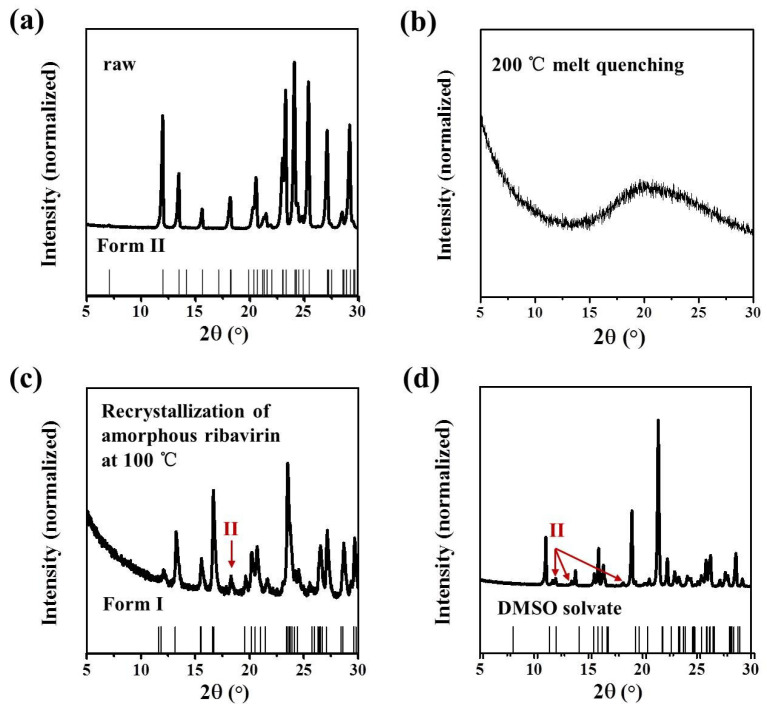
PXRD patterns of ribavirin (**a**) Form II; (**b**) amorphous phase; (**c**) Form I with Form II impurity; and (**d**) DMSO solvate with Form II impurity.

The commercial ribavirin used in this study was a white crystalline powder confirmed to be Form II by the PXRD pattern, with characteristic peaks at 7.1°, 12.0°, 13.5°, 15.6°, and 18.2° (Figure 3a). Amorphous ribavirin was prepared by a reported melt-quenching method [23], which is simple and robust. Commercial ribavirin powder was melted quickly on a hot plate at 200 °C to become a transparent glass under atmospheric air. Only a broad, diffuse diffraction peak around 20° was observed in the PXRD pattern of the transparent ribavirin glass (Figure 3b), which confirmed the phase purity of the amorphous ribavirin.

We did not obtain Form I as the main phase by solution methods, a result also reported in the literature [21]. Form I was synthesized with high reproducibility by the solid-state recrystallization of amorphous ribavirin (Figure 3c). Amorphous ribavirin was recrystallized on a 100 °C hot plate to give a crystalline product with characteristic PXRD peaks at 13.2°, 15.5°, and 16.6° consistent with Form I [24]. Although the main recrystallization product of amorphous ribavirin was Form I, the slight existence of Form II was also observed. Since Form I can transform spontaneously and irreversibly into Form II during the heating process (~70 °C) [25], it seems unavoidable to generate a minor proportion of Form II by this procedure.

Ribavirin DMSO solvate was synthesized via an anti-solvent method (Figure 3d), which is not easy to reproduce successfully. In our best results, DMSO solvate with a minor impurity of Form II was obtained with characteristic PXRD peaks at 11.1°, 11.7°, 13.8°, 16.0°, and 19.0° [24].

Low-frequency Raman spectroscopy (LFRS, below 300 cm^−1^) probes crystal vibrations and is a technique used to quickly distinguish polymorphs of an organic molecule [27]. In this study, complementary to the PXRD analyses, LFRS was used to distinguish ribavirin polymorphs. Although the Form I and DMSO solvate used in further experiments were not pure, the purities were high enough and the crystal sizes were large enough at the microscopic level to distinguish Form I or DMSO solvate from Form II in microregion confocal Raman spectroscopy.

**Figure 4:**
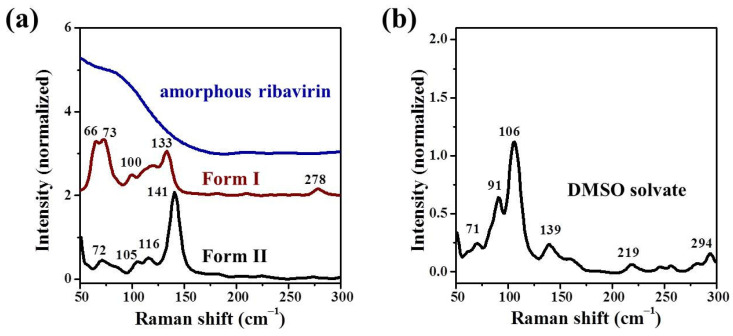
LFRS of ribavirin (**a**) Form II, amorphous phase, Form I and (**b**) DMSO solvate.

The typical LFRS bands of Form II with a 532 nm laser were at 72, 105, 116, and 141 cm^−1^ (Figure 4a), consistent with the reported 107, 118, and 144 cm^−1^ of Form II with a 785 nm laser [21]. The typical LFRS bands of Form I were at 66, 73, 100, and 133 cm^−1^. As would be expected, amorphous ribavirin does not have obvious characteristic LFRS bands.

The DMSO solvate had characteristic LFRS bands at 71, 91, 106, and 139 cm^−1^ (Figure 4b). Some of these bands (71, 106, and 139 cm^−1^) were similar to those of Form II (72, 105, and 141 cm^−1^), but their relative strengths were quite different, which also indirectly indicated that the DMSO solvate and Form II were two different phases. Thus, in the case of ribavirin, the use of LFRS alone is insufficient to distinguish the various ribavirin phases, especially Form II and DMSO solvate.

### 2.2. Selective Recrystallization of Amorphous Ribavirin

The amorphous phase has short-range order and lacks long-range order. The amorphous phase has high free–energy and will crystallize spontaneously into more stable solid forms. During the preparation process of ribavirin polymorphs, we found the selective recrystallization behavior of amorphous ribavirin to initially form Form I in a kinetic process. The short-range order of amorphous ribavirin includes molecular conformations and intermolecular interactions between adjacent ribavirin molecules. Mid-frequency Raman spectroscopy (MFRS) is suitable for studying the short-range orders of organic molecules in solutions or solids [3,4]. It can evaluate the similarity between phases by comparing the similarity of their spectra, whereby the higher the MFRS similarity between phases, the easier the selective recrystallization between them [28]. Thus, MFRS can be used to explain and predict selective crystallizations in theory.

**Figure 5:**
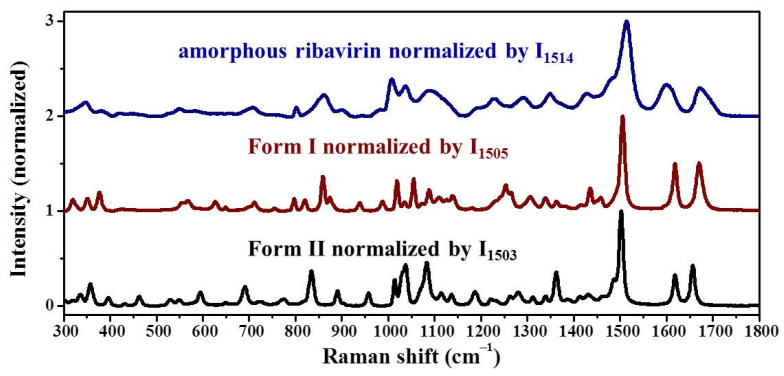
MFRS (300–1800 cm^−1^) of amorphous ribavirin, Form I, and Form II normalized by the intensities at 1514, 1505, and 1503 cm^−1^, separately. The strongest MFRS band is normalized.

The MFRS of amorphous ribavirin, Form I, and Form II normalized by the intensities at 1514, 1505 and 1503 cm^−1^, respectively, are shown in Figure 5 and Table 3. The MFRS of amorphous ribavirin, Form I, and Form II are very similar since their molecular compositions are the same. The Raman band widths of amorphous ribavirin are much wider than those of Form I and Form II, which is a consistent feature of the amorphous phase.

It is difficult to determine whether amorphous ribavirin is more similar to Form I or Form II by direct visual observation (Figure 5). The traditional characteristic Raman band comparison method is limited because the results are dependent on the choice of the characteristic Raman bands. For ribavirin (Table 3), if the Raman band at 1672 cm^−1^ of amorphous ribavirin is chosen, Form I (at 1670 cm^−1^) is more similar to amorphous ribavirin than Form II (at 1656 cm^−1^). However, if the Raman band at 1037 cm^−1^ of amorphous ribavirin is selected, Form II (at 1037 cm^−1^) is more similar to amorphous ribavirin than Form I (at 1054 cm^−1^). Furthermore, the Raman band at 1600 cm^−1^ of amorphous ribavirin cannot be used to compare the similarity to Form I or Form II (both at 1618 cm^−1^) [11]. The similarity orders of different phases should be derived from a comprehensive comparison to avoid subjective factors.

**Figure 6:**
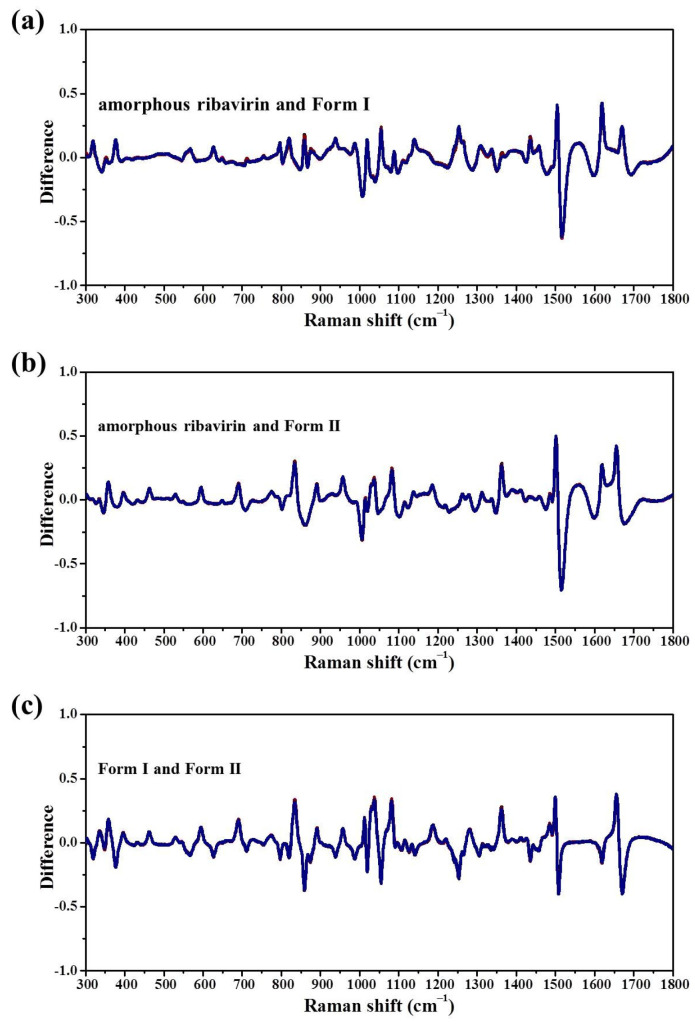
MFRDS between (**a**) amorphous ribavirin and Form I; (**b**) amorphous ribavirin and Form II; and (**c**) Form I and Form II. Each MFRDS contains 3 × 3 lines.

Mid-frequency Raman difference spectra (MFRDS) are used in our laboratory to establish the similarity orders of different phases of organic molecules and the relationships between selective recrystallization and the short-range orders of amorphous phases [11]. MFRDS between two spectra of amorphous ribavirin, Form I and Form II were obtained by subtracting the two normalized MFRS (Figure 6). The MFRDS between amorphous ribavirin and Form I (Figure 6a) appears slightly smoother than that between amorphous ribavirin and Form II (Figure 6b) in visual observation. This is still a subjective assessment, however.

The absolute deviation (a.d.) and standard deviation (s.d.) of MFRDS are used for a more objective description of the similarity order (Table 4). A smaller a.d. and s.d. indicate that the short-range orders of the two phases are more similar. Hence, for MFRDS between samples of the same phase, the a.d. and s.d. are very small (<5 and <10, respectively), which indicates the suitable reproducibility of the MFRS signals.

The a.d. and s.d. of MFRDS between amorphous ribavirin and Form I (0.062 and 0.095, respectively) are less than those between amorphous ribavirin and Form II (0.065 and 0.107), which indicates that the amorphous phase is more similar to Form I than Form II. The a.d. and s.d. of MFRDS between Form I and Form II are 0.060 and 0.095, close to those between amorphous ribavirin and Form I. In a detailed traditional Raman band comparison, of the 17 listed Raman bands of amorphous ribavirin, 11 are more similar to Form I than Form II, two Raman bands of Form I and Form II are the same, and the other four Raman bands of amorphous ribavirin are more similar to Form II (Table 3). The results also show that amorphous ribavirin and Form I have similar short-range orders, but the comparison process is elaborate and still somewhat subjective. We conclude that the selective crystallization process from amorphous ribavirin to Form I is caused by the similar short-range orders.

However, because the a.d. and s.d. of the MFRDS between amorphous ribavirin and Form I are only slighty smaller than those between amorphous ribavirin and Form II, the selective control is not very robust. This observation can explain why a pure Form I phase is difficult to obtain in the amorphous ribavirin recrystallization and solution crystallization processes.

### 2.3. Selective Recrystallization of Ribavirin Aqueous Solution

The solubilities of ribavirin are listed in various solvents in Table 5. Ribavirin is readily soluble (>10 wt%) in water, dimethylformamide (DMF), dimethylacetamide (DMA), and dimethyl sulfoxide (DMSO). Ribavirin is slightly soluble (<1 wt%) in methanol, ethanol, and acetone. The data unexpectedly show that the metastable Form I had poorer solubility than stable Form II.

The Raman spectra characterization process of solutions is nondestructive and requires no pretreatments. If the concentration of the ribavirin solution is less than 1 wt%, it is not easy to distinguish the Raman bands. The MFRS of ribavirin in methanol, ethanol, acetone, and ethyl acetate solutions only show the solvent signals (Figure S1) and the ribavirin solute signals cannot be obtained. 

**Figure 7:**
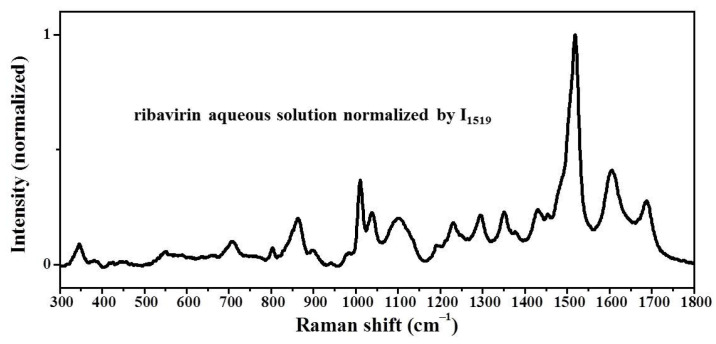
MFRS of ribavirin aqueous solution normalized by intensity at 1519 cm^−1^.

The LFRS of ribavirin saturated aqueous solution and saturated DMSO solution do not show obvious bands (Figure S2). This situation is similar to the LFRS of the amorphous phase, which is characterized by solution features and fails to provide valid information about the short-range orders. On the other hand, the MFRS of ribavirin saturated aqueous solution has a high signal to noise ratio (Figure 7). Since the Raman activity of water molecules is weak (Figure S3) and the aqueous solubility of ribavirin is high, the MFRS of the aqueous solution can be used for the ribavirin solute without differential treatment. The Raman bands of ribavirin aqueous solution are similar to those of amorphous ribavirin, except for the Raman band at 1689 cm^−1^ (Table 3). The shift between ribavirin aqueous solution at 1689 cm^−1^ and amorphous ribavirin at 1672 cm^−1^ is probably caused by the interaction between ribavirin and the water molecules, while the Raman peak of water is at ~1645 cm^−1^. The Raman band widths of ribavirin aqueous solution are also similar to those of the amorphous phase, which indicates that the dispersion degree of the short-range orders in the two phases is similar.

**Figure 8:**
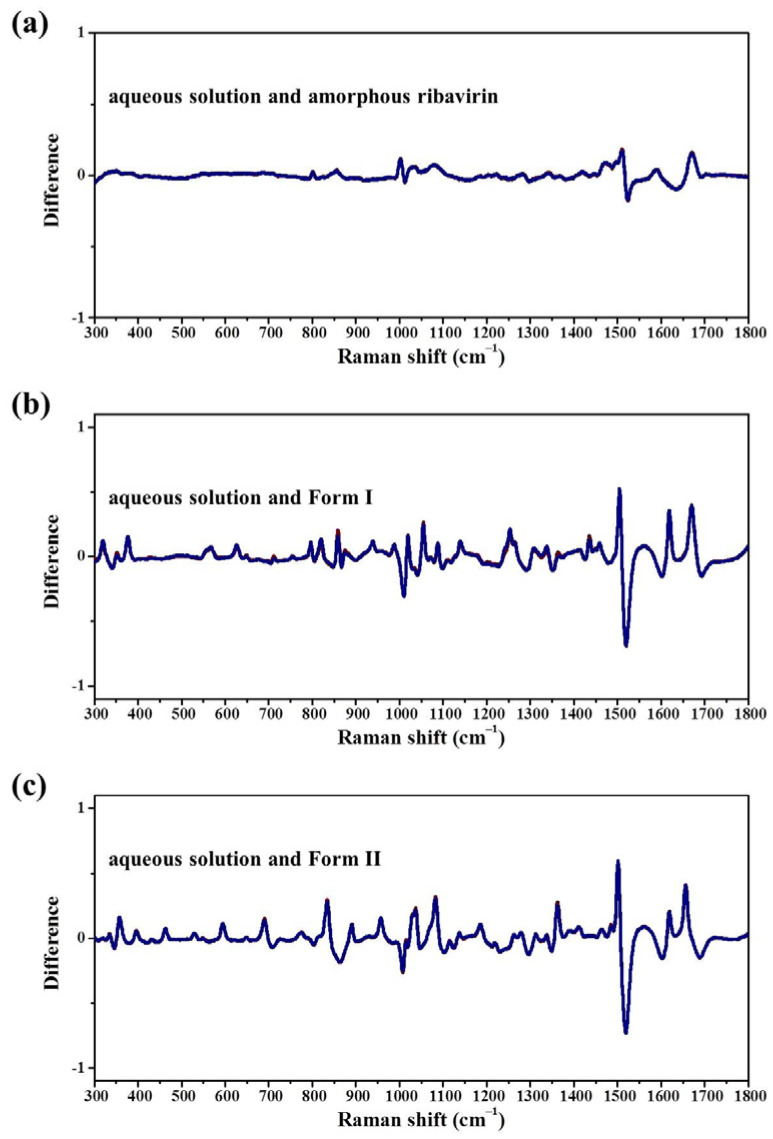
MFRDS between (**a**) ribavirin aqueous solution and amorphous ribavirin; (**b**) aqueous solution and Form I; and (**c**) aqueous solution and Form II. Each MFRDS contains 3 × 3 lines.

The results in Figure 8 and Table 6 show that the MFRDS curves between ribavirin aqueous solution and amorphous ribavirin are much smoother than those between the aqueous solution and Form I or Form II. Moreover, the a.d. and s.d. of the MFRDS between the aqueous solution and amorphous ribavirin (0.025 and 0.037) are much smaller than those between the aqueous solution and Form I (0.055 and 0.097) or Form II (0.060 and 0.107). These comparisons indicate that the short-range orders of ribavirin aqueous solution are similar to those of amorphous ribavirin, which is consistent with the common understanding [19].

**Figure 9:**
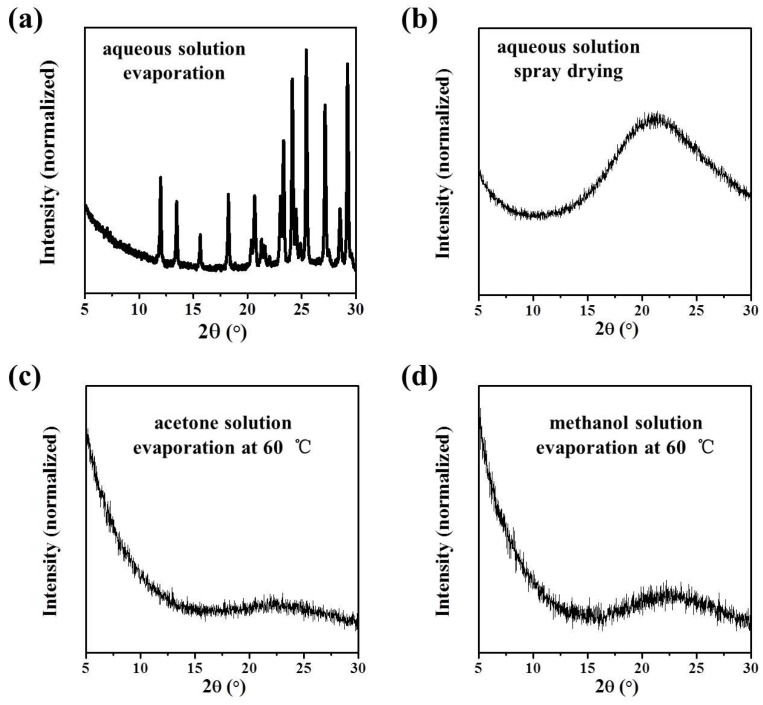
PXRD patterns of (**a**) evaporation product of 100 μL ribavirin–saturated aqueous solution at room temperature on an aluminum substrate; (**b**) spray–drying product of ribavirin aqueous solution (conditions are described in the Section 4); and evaporation products of 100 μL ribavirin–saturated (**c**) acetone; and (**d**) methanol solutions at 60 °C.

Thus, according to the MFRDS analysis, it is possible to obtain amorphous ribavirin from aqueous solution through kinetic control. Usually, the products of aqueous solution evaporation are Form II at room temperature (Figure 9a). The spray drying method was used to achieve the quick evaporation of water and provide amorphous ribavirin (Figure 9b). The inlet temperature was ~160 °C, which is lower than the melting temperature of ribavirin Form I and Form II [25]. It is also easy to obtain amorphous ribavirin via the quick evaporation of organic solvents such as acetone and methanol (Figure 9c,d). The preparation process of amorphous ribavirin via spray drying or quick evaporation has not been reported previously.

**Figure 10:**
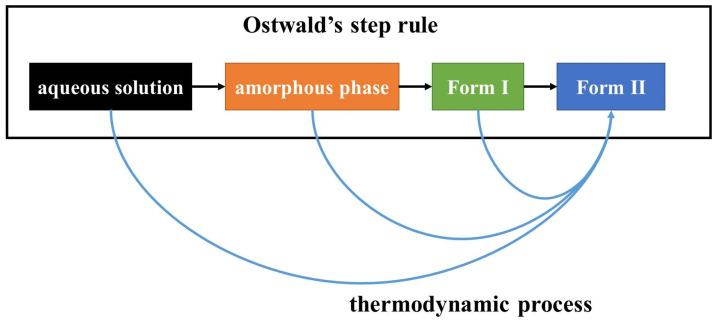
A schematic crystallization process of ribavirin aqueous solution. The process was divided into a kinetic process (Ostwald’s step rule) and a thermodynamic process. The kinetic process provides a metastable phase, while the thermodynamic process provides the most stable phase under given conditions.

The MFRDS results also indicate that ribavirin aqueous solution is slightly more similar to Form I than Form II. However, it has not been reported that Form I can be obtained from ribavirin aqueous solution, and it appears difficult to obtain in the other solvents in Table 1. In our experiments, we did not synthesize Form I via solution methods. The stability of Form I is between that of amorphous ribavirin and Form II [23]. Fast crystallization is beneficial to obtain amorphous ribavirin, and slow crystallization is beneficial for Form II. To obtain Form I from a solution phase directly, very fine control of the crystallization, including nucleation and stabilization of Form I should be realized simultaneously. However, a non-classical crystallization process from solution to amorphous phase to Form I could be easy to control in an anti-solvent or by evaporation methods (Figure 10).

### 2.4. Selective Recrystallization of Ribavirin DMSO Solution

**Figure 11:**
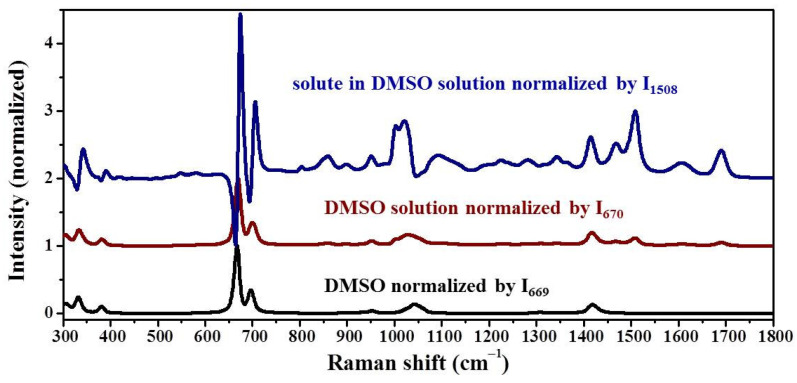
MFRS of DMSO normalized by intensity of the band at 669 cm^−1^, ribavirin DMSO solution normalized by intensity at 670 cm^−1^, and ribavirin solute in DMSO solution normalized by intensity at 1508 cm^−1^.

**Figure 12:**
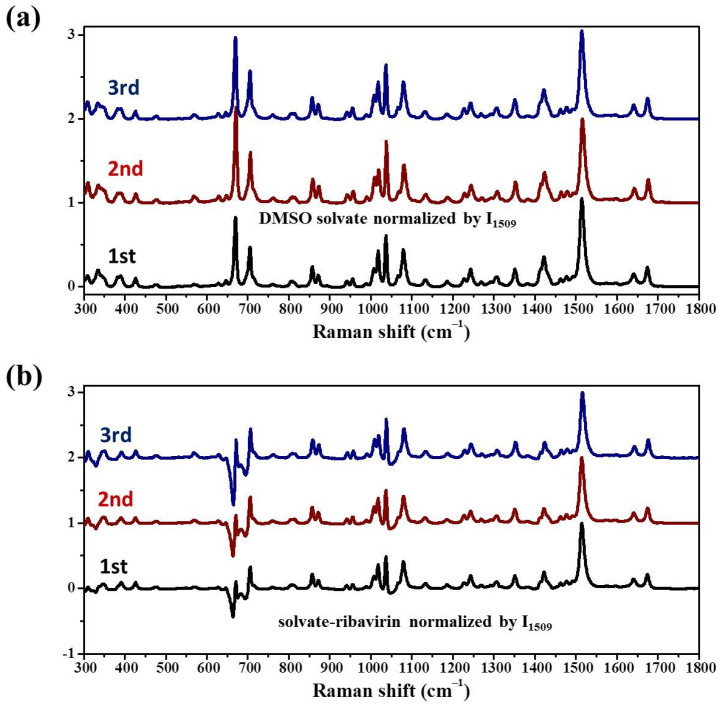
MFRS of (**a**) DMSO solvate with signals of DMSO solvent; and (**b**) DMSO solvate subtracting DMSO (ribavirin solvate).

The DMSO solvate is the only reported solvate of ribavirin, but it was not easy to prepare in our laboratory, indicating that it is not a stable phase. To understand why the DMSO solvate is so difficult to obtain, the MFRS of ribavirin solute (Figure 11) and DMSO solvate (Figure 12) were used in an MFRDS analysis. In contrast to water, DMSO solvent shows strong Raman bands and it is necessary to subtract these signals to obtain those of the solute ribavirin in DMSO solution, comprising signals of ribavirin itself and ribavirin interacting with DMSO. The Raman bands of the solute ribavirin strongly interact with the signals of DMSO, especially at 300–400 cm^−1^ and 600–800 cm^−1^.

The MFRS of the DMSO solvate showed DMSO Raman bands, which might have been caused by residual DMSO solvent or the DMSO molecules of the solvate. Considering that the reported mole fractions of ribavirin in DMSO and DMSO solvate are ~0.0728 and 1, respectively (Figure 12a), and the ratios of the band intensity at 1508 cm^−1^ and 667 cm^−1^ are ~0.12 in DMSO solution and ~1 in DMSO solvate, we propose that the DMSO signals are mainly related to DMSO solvate. To obtain the signals of ribavirin in DMSO solvate, differential spectrum processing was used to subtract the signals of DMSO (Figure 12b, ribavirin solvate) and obtain the signals of ribavirin.

**Figure 13:**
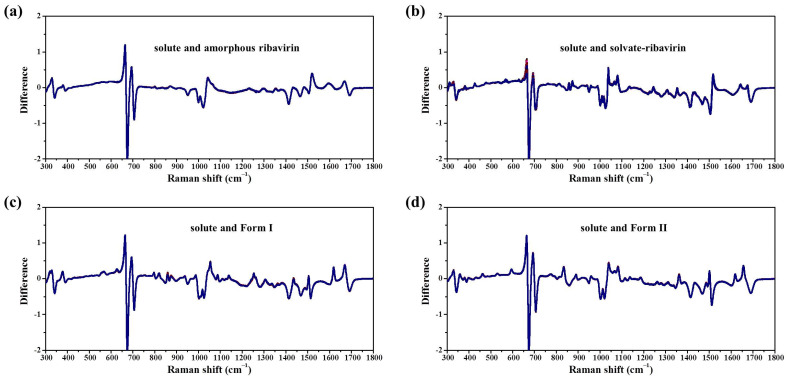
MFRDS of ribavirin solute in DMSO solution and (**a**) amorphous ribavirin; (**b**) solvate-ribavirin; (**c**) Form I; and (**d**) Form II.

MFRDS analysis was carried out to correlate the DMSO solution and phase outcomes (Figure 13, Table 7). Because the main bands of DMSO are in the range of 300–800 cm^−1^, the a.d. and s.d. of the MFRDS in the range of 800–1800 cm^−1^ are also given (Table 7). The MFRDS in the ranges of 300–1800 cm^−1^ and 800–1800 cm^−1^ show similar results. The phase most similar to ribavirin solute in DMSO is amorphous ribavirin (Figure 13a), with a.d. and s.d. notably smaller than the solute with ribavirin solvate, Form I, and Form II. The a.d. and s.d. between the solute and ribavirin solvate, Form I and Form II are similar (Figure 13b–d). Because the errors in the a.d. and s.d. are relatively large, it is not appropriate to give similarity orders. The crystallization process in DMSO solution is similar to that in aqueous solution, but an additional metastable phase (DMSO solvate) exists.

## 3. Discussion

### 3.1. Crystallization Selectivity in Solid and Solution Process

It is clear that the crystallization selectivity in the solid–solid process is more controllable than that in the solution–solid process. Form I can be obtained reproducibly in a solid–solid process without seeding, and we did not obtain Form I via solution methods. Thus, we provide a robust method to prepare the metastable phase via the amorphous intermediate phase.

### 3.2. Poor Repeatability of Form I and DMSO Solvate

The presence of solvents is not beneficial to the stability of metastable phases, which may explain why Form I or the DMSO solvate is not easy to obtain. It is possible to obtain metastable phases via the rapid removal of the solvent by spray drying, for example, which provided amorphous ribavirin from aqueous solution. Because the similarity order of Form I or DMSO solvate is between the amorphous phase and Form II, careful control of the crystallization rate or supersaturation is necessary in a CNT process. In addition, Form I or DMSO solvate should be stabilized by other factors to avoid transformation into the more stable phase. In an NCNT process, the amorphous phase should be unstable and able to transform into Form I or DMSO solvate consistent with the order of similarity.

It is unfortunate that we could not obtain the pure DMSO solvate phase in order to research its desolvation process. It is possible that this was because DMSO contains an amount of water sufficient to affect the crystallization process [32,33]. Based on the MFRDS results, a better method would involve the pure solvate. We were also unable to obtain inosine DMSO solvate in our previous work [11].

### 3.3. Shortcomings of MFRDS

The influence of the solvent cannot be avoided in the current method, especially when the Raman strength of the solvent is strong such as for DMSO. A more effective method should be developed to obtain consistent results with a lower margin of error.

## 4. Experiments

### 4.1. Materials

Ribavirin (C_8_H_12_N_4_O_5_, 98%, Form II) and ethyl acetate (C_4_H_8_O_2_, AR) were bought from shanghai Aladdin, China. dimethyl sulfoxide (DMSO, C_2_H_6_OS, AR) from Beijing Sinopharm, China. ethanol (C_2_H_6_O, AR) from Shanghai Hushi, China.

### 4.2. Polymorphs

Commercial ribavirin was provided as Form II. Quenched amorphous ribavirin was prepared by melting ribavirin powder on an aluminum substrate at 200 °C (DLAB HP380-Pro) for several seconds until the powder melted completely, and then cooling the liquid under atmospheric conditions. Spray dried amorphous ribavirin was obtained from 1 wt% ribavirin aqueous solution by spray drying (Shanghai Pilotech, Shanghai, China. YC-015, inlet 160 °C, 1.5–2 L·h^−1^). Ribavirin Form I was synthesized by heating amorphous ribavirin at 100 °C for several hours to ensure complete recrystallization of the amorphous phase. Ribavirin DMSO solvate was obtained by dissolving commercial ribavirin (0.5 g) in DMSO (2.5 mL), adding ethyl acetate (10 mL) and stirring overnight. The obtained DMSO solvate was filtered and washed with ethanol.

### 4.3. Characterization

Samples were characterized by powder X-ray diffraction (PXRD, Philips X’Pert Pro, Almelo, The Netherlands, Cu Kα, 40 kV, 30 mA, 5–30°, 4°·min^−1^) and confocal Raman spectroscopy (DXR3xi, Thermo Fisher Scientific, USA, 532 nm, 40 mW, 0.002–0.02 s, 1000 scans, 50–3400 cm^−1^, 50× objective lens). All Raman spectra were normalized in the range of 300–1800 cm^−1^ to the strongest band. MFRDS were carried out by the direct subtraction of two normalized MFRS and a polynomial fitting process to deduce the baseline.

## 5. Conclusions

In conclusion, the selective crystallization process from ribavirin aqueous solution to amorphous ribavirin and amorphous ribavirin to Form I was reported and explained by MFRDS analysis. The crystallization process of ribavirin solvate from ribavirin DMSO solution is difficult to control due to competitive transformations into amorphous ribavirin or Form I. The similarity analysis of the short-range orders based on MFRDS has provided a basis to prepare metastable phases, amorphous ribavirin, ribavirin Form I, and DMSO solvate, which is an important development in polymorph control for the laboratory and for industrial production.

## Figures and Tables

**Figure 1:**
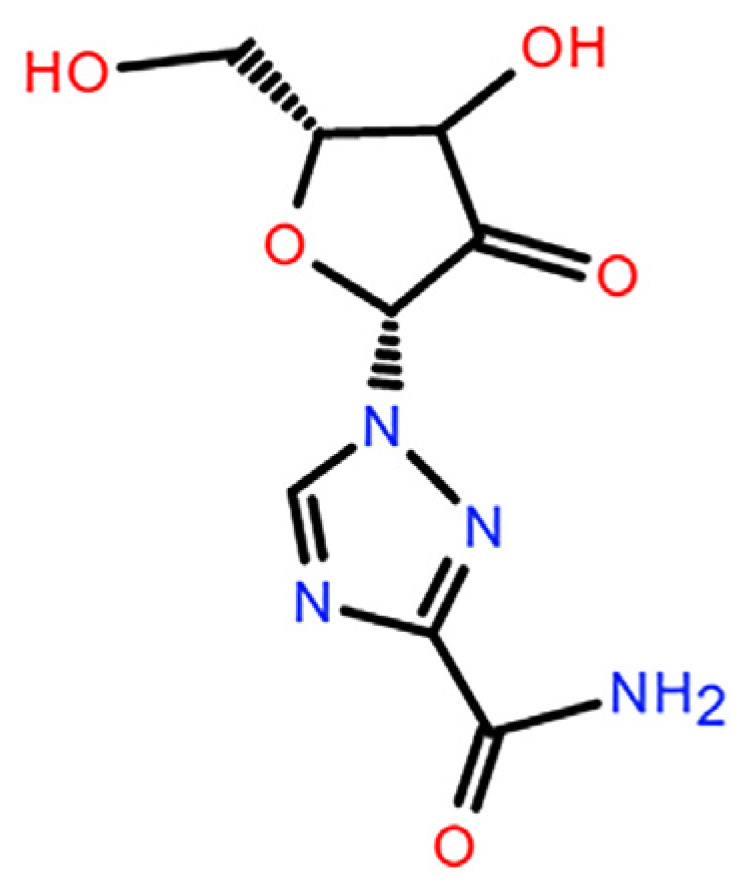
Molecular structure of ribavirin.

**Table 1:** Crystal cell parameters of ribavirin Form II, Form I and DMSO solvate.

Cell Parameters	Form II [24]	Form I [24]	DMSO Solvate [23]
CCDC number	VIRAZL01	VIRAZL	-
Space group	P2_1_2_1_2_1_	P2_1_2_1_2_1_	P2_1_
a (Å)	25.03	14.86	8.26
b (Å)	7.72	7.51	7.73
c (Å)	5.29	8.79	11.80
α (°)	90	90	90
β (°)	90	90	105.5
γ (°)	90	90	90

**Table 2:** The methods to prepare ribavirin Form II, Form I, amorphous phase and DMSO solvate.

Phase	Method	Ref.
Form II	commercial and clinical materials	
	recrystallization in methanol from 50 °C to room temperature	[23]
	recrystallization in water from 80 °C to 25 °C	[25]
Form I	recrystallization in DMF–acetone (1:3) from 65 °C to room temperature	[23]
	rapid evaporation of methanol solution at 60 °C with isolated Form I crystals as seeds	[25]
	ball milling	[25]
	anti-solvent crystallization, DMA, and n-butanol	[26]
	recrystallization of amorphous phase at 100 °C	this work
amorphous phase	quenching melt from 170 °C to 10 °C	[23]
	cryogenic milling	[25]
	spray drying of aqueous solution	this work
DMSO solvate	recrystallization in DMSO–ethyl acetate (1:4) from 70 °C to room temperature	[23]
	anti-solvent crystallization, DMSO, and alcohol solvents	[26]

**Table 3:** Characteristic MFRS bands (cm^−1^) of Form II, Form I, amorphous ribavirin, DMSO solvate, aqueous solution, and DMSO solution.

Form II	Form I	Amorphous	DMSO Solvate	Form II Solution	Form I Solution
1656	1670	1672	1674	1689	1691
1618	1618	1600	1641	1604	1609
1502	1505	1514	1514	1519	1509
1432	1435	1429	1422	1430	
1339	1339	1348	1351	1350	1344
1312	1307	1290	1307 + 1293	1294	1282
1263	1253	1228	1243 + 1228	1229	1226
1083	1088	1087	1079 + 1067	1101	
1037	1054	1037	1037	1038	
1013	1019	1008	1018 + 1008	1011	1004
890	859	862	872 + 857	862	860
834	820	802	807	803	805
690	712	708		707	
549	567	549	568 + 548	551	
432	426	421	426		
396	377	382	390 + 384	384	
358	351	346	335	345	

**Table 4:** MFRDS results between two phases of amorphous ribavirin, Form I and Form II.

300–1800 cm^−1^	a.d. × 10^3^	s.d. × 10^3^
self- amorphous ribavirin	2.2 (0.1)	2.7 (0.2)
self- Form I	4.8 (2.8)	6.9 (4.2)
self-Form II	2.7 (0.6)	4.5 (1.3)
amorphous ribavirin and Form I	62.2 (1.3)	95.2 (1.1)
amorphous ribavirin and Form II	64.5 (0.2)	107.2 (0.2)
Form I and Form II	59.8 (1.1)	95.1 (1.0)

**Table 5:** Ribavirin solubility in different solvents at 298.15 K.

Solvents	Form	Mole Fraction Solubility	g per 100 g Solvent	Ref.
water	Form II	10.9 × 10^−3^	14.9	[26,29]
	Form I	9.97 × 10^−3^	13.7	[26]
	Form II	14.41 × 10^−3^	19.8	[30]
	Form II	14.42 × 10^−3^	19.8	[31]
methanol	Form II	1.278 × 10^−3^	0.975	[30]
	Form II	0.8652 × 10^−3^	0.660	[31]
ethanol	Form II	0.1575 × 10^−3^	0.084	[31]
acetone	Form II	0.0636 × 10^−3^	0.027	[31]
DMF	Form II	44.4 × 10^−3^	15.5	[26]
DMA	Form II	74.3 × 10^−3^	22.5	[26]
DMSO	Form II	65.8853 × 10^−3^	22.0	[31]

**Table 6:** MFRDS results of ribavirin aqueous solution with amorphous ribavirin, Form I, and Form II.

300–1800 cm^−1^	a.d. × 10^3^	s.d. × 10^3^
self- amorphous ribavirin	2.0 (0.3)	2.5 (0.4)
aqueous solution and amorphous ribavirin	24.6 (0.2)	37.4 (0.2)
aqueous solution and Form I	55.3 (1.4)	97.1 (0.9)
aqueous solution and Form II	59.7 (0.2)	107.0 (0.2)

**Table 7:** MFRDS results of ribavirin solute in DMSO solution with amorphous ribavirin, Form I, Form II, and ribavirin solvate.

Range	300–1800 cm^−1^		800–1800 cm^−1^	
a. d. and s. d.	a.d. × 10^3^	s.d. × 10^3^	a.d. × 10^3^	s.d. × 10^3^
self-solute	6.2 (1.0)	11 (3)	5.7 (1.8)	6.7 (1.8)
Self–solvate–ribavirin	14 (6)	28 (14)	13 (6)	23 (12)
solute with solvate–ribavirin	147 (2)	237 (4)	135 (2)	191 (3)
solute with amorphous phase	115 (2)	221 (4)	91 (2)	133 (2)
solute with Form I	149 (2)	247 (4)	134 (2)	187 (2)
solute with Form II	151 (2)	250 (4)	138 (2)	191 (2)
solute with aqueous solution	132 (2)	231 (3)	116 (2)	151 (2)

## Data Availability

Not applicable.

## Supplementary Materials

The following supporting information can be downloaded at: https://www.mdpi.com/article/10.3390/molecules28176320/s1, Figure S1. MFRS of solvents and ribavirin saturated solution. (a) acetone and ribavirin acetone solution, (b) methanol and ribavirin methanol solution, (c) ethanol and ribavirin ethanol solution, and (d) ethyl acetate and ribavirin ethyl acetate; Figure S2. LFRS of ribavirin (a) aqueous solution and (b) DMSO solution. Figure S3. MFRS of water.

## References

[1] Higashi K., Ueda K., Moribe K. (2017). Recent progress of structural study of polymorphic pharmaceutical drugs. Adv. Drug Deliv. Rev..

[2] Yao C., Zhang S., Wang L., Tao X. (2022). Recent Advances in Polymorph Discovery Methods of Organic Crystals. Cryst. Growth Des..

[3] Davey R.J., Schroeder S.L., ter Horst J.H. (2013). Nucleation of organic crystals—A molecular perspective. Angew. Chem. Int. Ed. Engl..

[4] Li X., Wang J., Wang T., Wang N., Zong S., Huang X., Hao H. (2021). Molecular mechanism of crystal nucleation from solution. Sci. China Chem..

[5] Gataullina K., Buzyurov A., Ziganshin M., Padnya P., Stoikov I., Schick C., Gorbatchuk V. (2019). Using fast scanning calorimetry to detect guest-induced polymorphism by irreversible phase transitions in the nanogram scale. Crystengcomm.

[6] Corvis Y., Wurm A., Schickb C., Espeaua P. (2015). New menthol polymorphs identified by flash scanning calorimetry. CrystEngComm.

[7] Cruz-Cabeza A.J., Reutzel-Edens S.M., Bernstein J. (2015). Facts and fictions about polymorphism. Chem. Soc. Rev..

[8] Shi Q., Chen H., Wang Y., Xu J., Liu Z., Zhang C. (2022). Recent advances in drug polymorphs: Aspects of pharmaceutical properties and selective crystallization. Int. J. Pharm..

[9] Gentili D., Gazzano M., Melucci M., Jonesb D., Cavallini M. (2019). Polymorphism as an additional functionality of materials for technological applications at surfaces and interfaces. Chem. Soc. Rev..

[10] Gabdulkhaev M., Ziganshin M., Buzyurov A., Schick C., Solovieva S., Popova E., Gubaidullin A., Gorbatchuk V. (2020). Smart control of calixarene polymorphic states. CrystEngComm.

[11] Chen F., Yang C., Cheng X., Fan Y., Chen X., Ren S., Xue R. (2022). Explanation for the selective crystallization from inosine solutions using mid-frequency Raman difference spectra analysis. RSC Adv..

[12] Gur D., Politi Y., Sivan B., Fratzl P., Weiner S., Addadi L. (2013). Guanine-based photonic crystals in fish scales form from an amorphous precursor. Angew. Chem. Int. Ed..

[13] Chen F., Wu B., Elad N., Gal A., Liu Y., Ma Y., Qi L. (2019). Controlled crystallization of anhydrous guanine β nano-platelets via an amorphous precursor. CrystEngComm.

[14] Sun S., Chevrier D.M., Zhang P., Gebauer D., Colfen H. (2016). Distinct Short-Range Order Is Inherent to Small Amorphous Calcium Carbonate Clusters (<2 nm). Angew. Chem. Int. Ed. Engl..

[15] Chen F., Ma Y., Wang Y., Qi L. (2018). A novel tautomeric polymorph of anhydrous guanine and its reversible water harvesting property. Cryst. Growth Des..

[16] Gillon A.L., Davey R.J., Storey R., Feeder N., Nichols G., Dent G., Apperley D.C. (2005). Solid state dehydration processes:  Mechanism of water loss from crystalline inosine dihydrate. J. Phys. Chem. B.

[17] Sosso G.C., Chen J., Cox S.J., Fitzner M., Pedevilla P., Zen A., Michaelides A. (2016). Crystal Nucleation in Liquids: Open Questions and Future Challenges in Molecular Dynamics Simulations. Chem. Rev..

[18] De Yoreo J.J., Gilbert P.U., Sommerdijk N.A., Penn R.L., Whitelam S., Joester D., Zhang H., Rimer J.D., Navrotsky A., Banfield J.F. (2015). Crystallization by particle attachment in synthetic, biogenic, and geologic environments. Science.

[19] Chen X., Chen X., Sun H., Li X., Ren S., Jin C., Jin H., Chen F., Xue R. (2023). Explanations for the Selective Evaporative Crystallization of Simvastatin from Different Solutions Using Mid-Frequency Raman Difference Spectra. Appl. Spectrosc..

[20] Liu L., Guo S., Che C., Su Q., Zhu D., Hai X. (2022). Improved HPLC method for the determination of ribavirin concentration in red blood cells and its application in patients with COVID-19. Biomed. Chromatogr..

[21] Davis M.P., Korter T.M. (2022). Low-Frequency Vibrational Spectroscopy and Quantum Mechanical Simulations of the Crystalline Polymorphs of the Antiviral Drug Ribavirin. Mol. Pharm..

[22] Miles D.L., Miles D.W., Redington P., Eyring H. (1976). Theoretical studies of the conformational properties of ribavirin. Proc. Natl. Acad. Sci. USA.

[23] Cheng X., Song J., Zhang L., Yang S., Shi Y., Du G., Lv Y. (2013). Polymorphism and Pharmacokinetic Research of Ribavirin. Chin. Pharm. J..

[24] Prusiner P., SUNDARALINGAM M. (1976). The crystal and molecular structures of two polymorphic crystalline forms of virazole (1-β-D-ribofuranosyl-1,2,4-triazole-3-carboxamide). A New Synthetic Broad Spectrum Antiviral Agent. Acta Cryst..

[25] Vasa D.M., Wildfong P.L.D. (2017). Solid-state transformations of ribavirin as a result of high-shear mechanical processing. Int. J. Pharm..

[26] Feng Y., Hao H., Chen Y., Wang N., Wang T., Huang X. (2022). Enhancement of Crystallization Process of the Organic Pharmaceutical Molecules through High Pressure. Crystals.

[27] Jantschke A., Pinkas I., Hirsch A., Elad N., Schertel A., Addadi L., Weiner S. (2019). Anhydrous β-guanine crystals in a marine dinoflagellate: Structure and suggested function. J. Struct. Biol..

[28] Fan Y., Liang C., Li Y., Xiao W., Niu Y., Jin H., Xue R., Chen F. (2023). Explanation and prediction for the selective crystallization of boscalid by mid-frequency Raman difference spectra analysis. CrystEngComm.

[29] Feng Y., Hao H., Tian B., Chen K., Wang N., Wang T., Huang X. (2022). Influence of high pressure on the solubility of ribavirin in six pure solvents from 283.15 to 323.15 K. J. Chem. Thermodyn..

[30] Li X., Cong Y., Li W., Yan P., Zhao H. (2017). Thermodynamic modelling of solubility and preferential solvation for ribavirin (II) in co-solvent mixtures of (methanol, n-propanol, acetonitrile or 1,4-dioxane) + water. J. Chem. Thermodyn..

[31] Li Q., Zhou L., Zhang S., Shi K., Dai J., Wang H., Yin Q., Wang Z. (2021). Measurement and Correlation of the Solubility and Thermodynamic Properties of Ribavirin(II) in Nine Pure Solvents and (1-Propanol + Water) Binary Solvents. J. Chem. Eng. Data.

[32] Morozova A., Ziganshina S., Kudryavtseva E., Kurbatova N., Savostina L., Bukharaev A., Ziganshin M. (2022). Water admixture triggers the self-assembly of the glycyl-glycine thin film at the presence of organic vapors. Colloids Surf. A Physicochem. Eng. Asp..

[33] Li Q., Ma H., Wang A., Jia Y., Dai L., Li J. (2015). Self-Assembly of Cationic Dipeptides Forming Rectangular Microtubes and Microrods with Optical Waveguiding Properties. Adv. Opt. Mater..

