# Supplementary material for: Crystallization Selectivity of Ribavirin Solution and Amorphous Phase
Source: Molecules. 2023 Aug 29;28(17):6320. doi: 10.3390/molecules28176320 (PMC10488721; doi:10.3390/molecules28176320)
Supplement: Supplementary file 1 [file molecules-28-06320-s001.zip › molecules-2507772-supplementary.pdf]

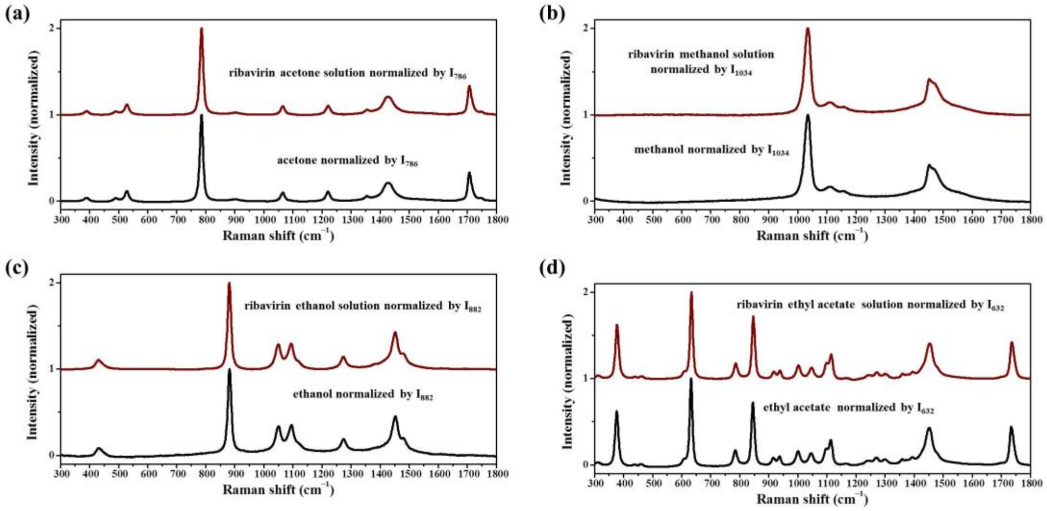

Figure S1. MFRS of solvents and ribavirin saturated solution. (a) acetone and ribavirin acetone solution, (b) methanol and ribavirin methanol solution, (c) ethanol and ribavirin ethanol solution, and (d) ethyl acetate and ribavirin ethyl acetate.

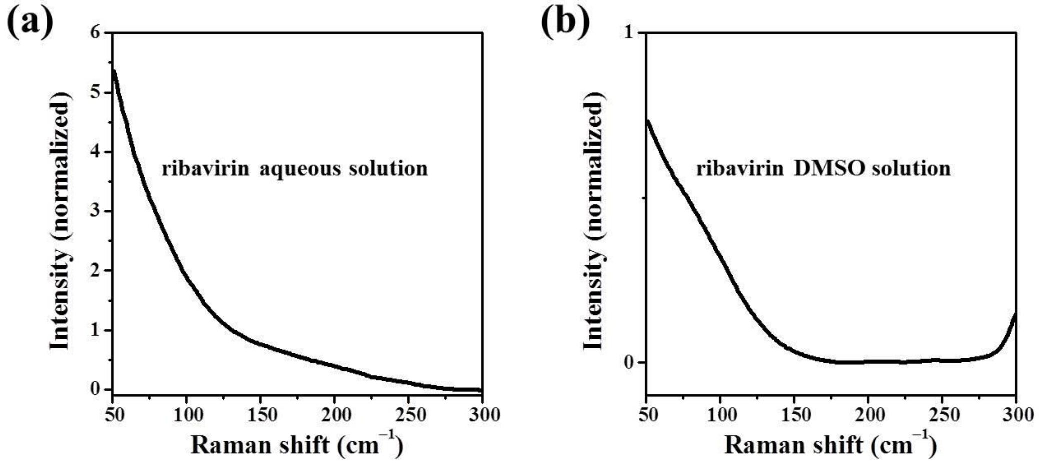

Figure S2. LFRS of ribavirin (a) aqueous solution and (b) DMSO solution.

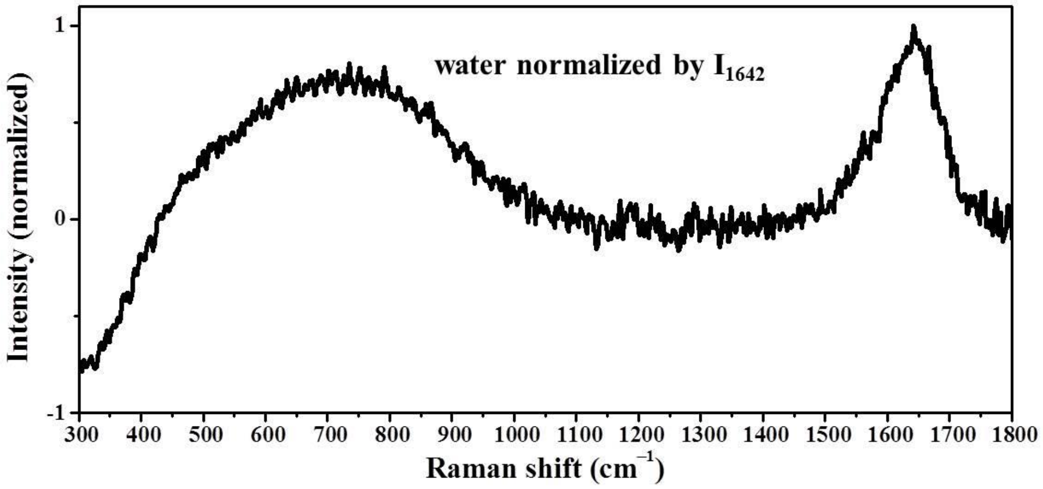

Figure S3. MFRS of water.
